# Epidermal growth factor receptor modulation for neural repair: Implications for neurodegenerative disease therapy

**DOI:** 10.3389/fnmol.2026.1891964

**Published:** 2026-07-29

**Authors:** Abdulbasit Amin, Marina Badenes

**Affiliations:** 1 Gulbenkian Institute for Molecular Medicine, Lisbon, Portugal; 2 Department of Physiology, Faculty of Basic Medical Sciences, University of Ilorin, Ilorin, Nigeria; 3 Research in Veterinary Medicine (I-MVET), Lusófona University - Lisbon University Centre, Lisbon, Portugal; 4 Veterinary and Animal Research Centre (CECAV), Faculty of Veterinary Medicine, Lusófona University - Lisbon University Centre, Lisbon, Portugal

**Keywords:** EGFR, EGFR ligands, nervous system, neural repair, neural therapy, neurodegenerative diseases

## Abstract

The epidermal growth factor receptor (EGFR; ErbB1/HER1) is a receptor tyrosine kinase that regulates cell proliferation, survival, differentiation, and tissue repair. In the nervous system, EGFR is expressed in neural progenitors, astrocytes, oligodendrocyte precursor cells, and neuronal populations, where its functions are context dependent. EGFR signaling contributes to neural regeneration by promoting progenitor proliferation, neuronal survival, neurogenesis, and remyelination following injury. However, sustained or excessive EGFR activation can drive reactive astrogliosis, neuroinflammation, glial scar formation, and neurotoxicity. Emerging evidence suggests that transient, regulated EGFR activation supports neural repair, whereas chronic or dysregulated signaling may contribute to neurodegeneration. These apparently opposing effects likely reflect differences in timing, duration, cellular context, ligand availability, and downstream signaling pathways engaged by EGFR activation, rather than inherently contradictory biological functions. In experimental models of Parkinson’s disease, Alzheimer’s disease, and Multiple sclerosis-like conditions, EGFR modulation has shown therapeutic potential, although the mechanisms remain incompletely understood. While EGFR ligands often exert neurotrophic and pro-remyelinating effects, disease-associated EGFR activation may promote maladaptive signaling pathways. In this review, we summarize current knowledge of EGFR signaling in neural repair and neurodegenerative diseases, discuss the context-dependent roles of this pathway, and highlight therapeutic strategies. We further propose a conceptual framework in which EGFR functions as a context-dependent signaling hub, with its outcomes determined by the spatiotemporal regulation of receptor activation. Although challenges remain, including optimal timing, dosing, and safety considerations, preclinical evidence suggests that modulation of EGFR signaling may be a therapeutic approach to promote neural repair while limiting neurodegenerative pathology.

## Graphical abstract

**Figure:**
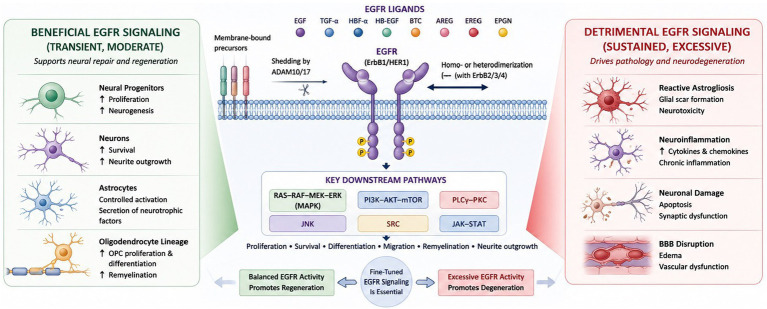
EGFR Signaling: a double-edged sword in the nervous system. *Context, Intensity and Duration Determine Outcomes in Neural Repair and Neurodegeneration* EGFR signaling exerts dual and context-dependent effects in the nervous system. Moderate and transient EGFR activation promotes neural repair and regeneration by enhancing neural progenitor proliferation, neurogenesis, neuronal survival, neurite outgrowth, astrocyte-mediated neurotrophic support, oligodendrocyte precursor cell expansion, and remyelination. In contrast, sustained or excessive EGFR activation contributes to pathological processes including reactive astrogliosis, neuroinflammation, glial scar formation, neuronal damage, and blood–brain barrier disruption. EGFR signaling is initiated by ligand binding, including EGF, TGF-α, HB-EGF, BTC, AREG, EREG, and EPGN, leading to receptor dimerization and activation of downstream pathways such as RAS/RAF/MEK/ERK-MAPK, PI3K/AKT/mTOR, PLCγ/PKC, JNK, SRC, and JAK/STAT. Dysregulated EGFR signaling has been implicated in neural injury and neurodegenerative disorders including Parkinson’s disease, Alzheimer’s disease, and multiple sclerosis. Therapeutic modulation of EGFR signaling, through EGFR inhibitors, ligand-specific targeting, or pathway-selective interventions, may therefore represent a promising strategy for promoting neural repair while limiting neurodegeneration.

## 1 Introduction

### 1.1 EGFR signaling in the nervous system

**Figure 1:**
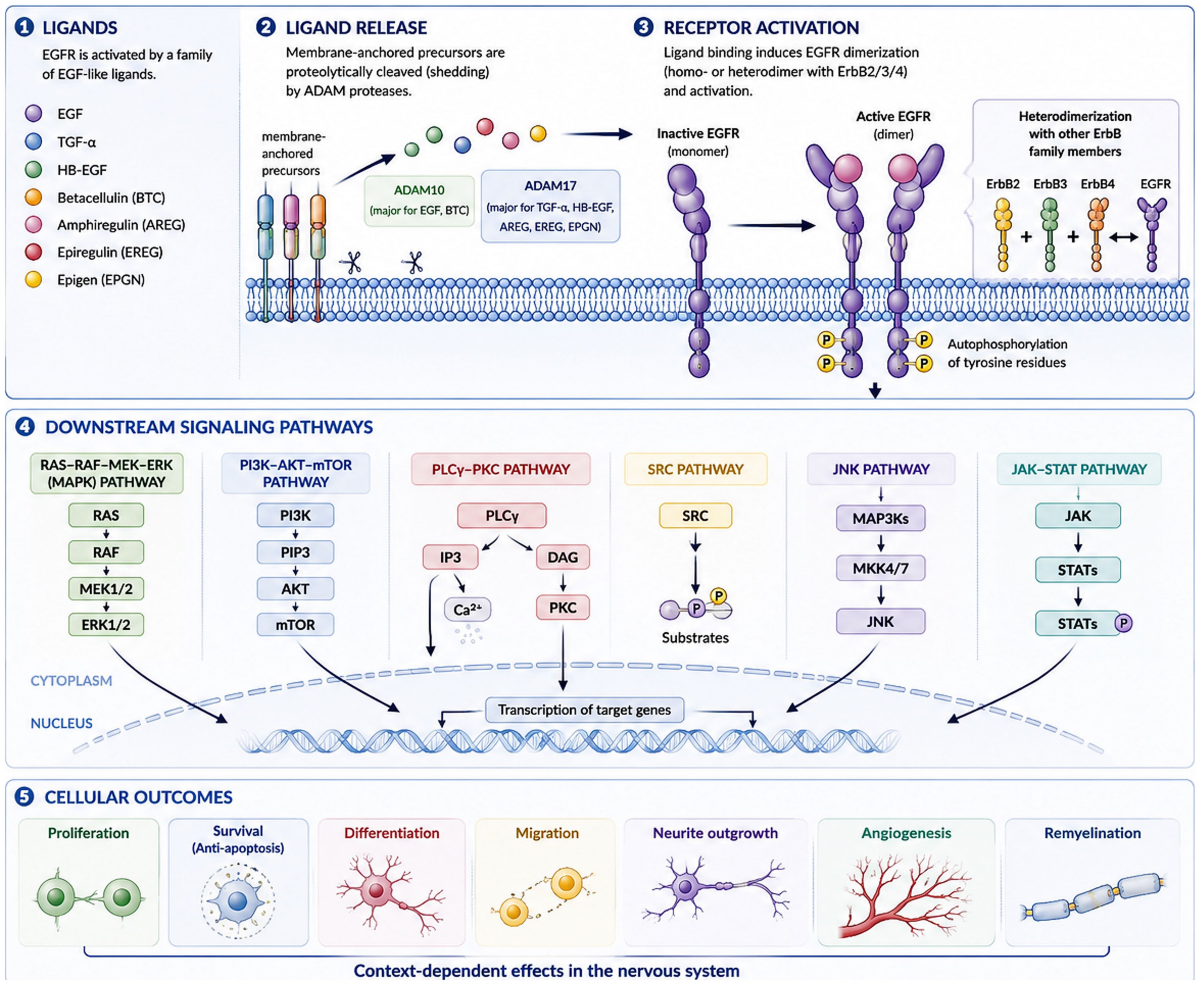
EGFR signaling pathway and its downstream cellular effects in the nervous system. EGFR signaling is initiated by binding of EGF-like ligands, including EGF, TGF-α, HB-EGF, betacellulin (BTC), amphiregulin (AREG), epiregulin (EREG), and epigen (EPGN). These ligands are generated through proteolytic cleavage (“shedding”) of membrane-bound precursors primarily by the metalloproteases ADAM10 and ADAM17. Ligand binding induces EGFR homo- or heterodimerization with other ErbB family members (ErbB2/3/4), resulting in receptor autophosphorylation and activation of downstream signaling cascades. Major downstream pathways include the RAS/RAF/MEK/ERK–MAPK, PI3K/AKT/mTOR, PLCγ/PKC, SRC, JNK, and JAK/STAT pathways, which regulate transcriptional programs involved in proliferation, survival, differentiation, migration, neurite outgrowth, angiogenesis, and remyelination. EGFR signaling is tightly regulated through receptor internalization, lysosomal degradation, dephosphorylation by protein tyrosine phosphatases, and negative feedback mechanisms. In neural repair contexts, EGFR activation engages pro-survival and regenerative pathways, principally the PI3K/AKT and MAPK/ERK cascades, which promote neuronal survival, neural progenitor proliferation, oligodendrocyte lineage expansion, remyelination, and tissue repair. In contrast, sustained or dysregulated EGFR activation can promote pathological responses through prolonged MAPK/ERK activity and activation of JAK/STAT signaling, contributing to neuroinflammation, astrogliosis, impaired proteostasis, and, in some contexts, excitotoxic injury. Thus, the functional consequences of EGFR signaling are determined not simply by receptor activation but by the cellular context and the duration, magnitude, and downstream integration of signaling. This figure was generated using BioRender.

The epidermal growth factor receptor (EGFR, also known as ErbB1/HER1) is a transmembrane receptor tyrosine kinase of the ErbB family that regulates cell proliferation, survival, migration, and differentiation (Cohen et al., 1980; Shaban et al., 2023). EGFR is activated by several ligands, including epidermal growth factor (EGF), transforming growth factor-*α* (TGF-α), heparin-binding EGF-like growth factor (HB-EGF), betacellulin (BTC), amphiregulin (AREG), epiregulin (EREG), and epigen (EPGN), which are released from membrane-bound precursors mainly through ADAM10/17-mediated proteolytic cleavage (Adrain and Badenes, 2024; Duffy et al., 2011; Rosenbaum and Saftig, 2024), (Saad and Jenkins, 2024). Ligand binding induces EGFR homo- or heterodimerization with other ErbB receptors, activating downstream signaling pathways including RAS/RAF/MEK/ERK–MAPK, PI3K/AKT/mTOR, PLCγ/PKC, SRC, JNK, and JAK/STAT cascades, which collectively regulate diverse cellular functions in the nervous system (Rosenkranz and Slastnikova, 2020, Romano and Bucci, 2020; Figure 1). These pathways are involved in neuronal repair and in the pathobiology of several neurodegenerative diseases, as summarized in Tables 1, 2, respectively. The biological outcome of EGFR activation is determined by multiple factors, including ligand availability, signal intensity and duration, receptor dimerization partners, and the responding cell type. Consequently, EGFR signaling can support either tissue repair or pathological remodeling depending on the physiological or disease context. This context-dependent nature of EGFR signaling provides the central framework of this review and offers a mechanistic basis for understanding why both EGFR activation and inhibition have produced beneficial effects in different experimental models of neurological disease.

**Table 1:** EGFR downstream pathways in neural repair.

Condition	Pathway	Model	Role	**Citation**
ON injury	RAS/ ERK	*In vivo:* ON crush (mouse); ON temporary clamp (rat). *In vitro:* primary neuron models (rat).	Dual role. EGFR-dependent activation mediates myelin- and CSP-induced axon regeneration inhibition. ERK activation downstream of pro-survival/regenerative signals promotes RGC survival and axon regeneration. SOE: Moderate.	Fujita et al. (2013), Walker et al. (2013), and Koprivica et al. (2005)
	PI3K/ AKT	*In vivo*: ON crush (goldfish); ON crush/temporary clamp (mouse, rat). *In vitro:* retinal explants (goldfish); primary RPE cells (mouse).	Protective. Activation enhances RGC survival and neurite regrowth. SOE: High.	Koriyama et al. (2007), Walker et al. (2013), Schuld et al. (2015), and Wen et al. (2019)
	JAK/ STAT	*In vivo:* optic nerve crush (mouse, rat); lens injury (rat); optic nerve transection (zebrafish). *In vitro:* retinal tissue and retinal cell assays (rat).	Protective. STAT3 signaling promotes RGC survival and axon regeneration. STAT3 is required for the initiation of the Müller glial reactive (astrogliosis-like) response, which supports regenerative/neuroprotective inflammation after optic nerve injury. SOE: High.	Wen et al. (2019), Kirsch et al. (2010), Venanzi et al. (2025), Lee et al. (2024), Sun et al. (2017), and Chen et al. (2022)
Glaucoma	RAS/ ERK	*In vivo:* microbead-mediated IOP elevation (mouse); Human patients’ eyes.	Mainly pathogenic. Chronic activation in glial cells promotes neuroinflammation and RGC degeneration, whereas transient neuronal ERK activation may support RGC survival under stress. SOE: Moderate.	Palanivel et al. (2024), Zong et al. (2025), and Tezel et al. (2003)
	PI3K/ AKT	*In vivo:* microbead-induced ocular hypertension and episcleral vein occlusion (mouse); hypertonic saline-induced ocular hypertension (rat). *In vitro:* primary RGC cultures (mouse) and ON head astrocytes (human). Human patients’ eyes.	Protective. Promotes RGC survival and inhibits apoptosis. SOE: High.	Palanivel et al. (2024), Wang et al. (2025a), Nie et al. (2018), and Husain et al. (2017)
	JAK/ STAT	*In vivo:* microbead-induced ocular hypertension (mouse, rat); saline-induced ocular hypertension (mouse). *In vitro:* primary Müller glia and RGC cultures. Human patients’ eyes.	Predominantly protective. STAT3 signaling in Müller glia and astrocytes promotes a neuroprotective reactive gliosis that supports RGC survival, although prolonged activation may also contribute to inflammatory responses. SOE: High.	Zong et al. (2025), Sun et al. (2017), Li Q. et al. (2021), Yang et al. (2011), and Salkar et al. (2024)
Epilepsy	RAS/ ERK	*In vivo:* kainic acid-induced epilepsy (mouse). *In vitro:* HT22 mouse hippocampal neurons; SH-SY5Y human neuroblastoma cells.	Predominantly pathogenic. Sustained activation following seizures promotes excitotoxic neuronal death and contributes to epileptogenesis. SOE: High.	Gan et al. (2022) and Gautam et al. (2021)
	PI3K/ AKT	*In vivo:* neuronal PTEN knockout (mouse); kainic acid-induced epilepsy (rat); TSC and FCD (mouse).	Context-dependent (predominantly pathogenic via mTOR). PI3K/Akt/mTOR chronic activation promotes neuronal hyperexcitability, epileptogenesis, and epilepsy-associated cortical malformations. AKT signaling also supports neuronal survival. SOE: High.	Gautam et al. (2021), Sumadewi et al. (2023), Ravizza et al. (2024), Prina et al. (2026), Sosanya et al. (2015), Hsieh et al. (2020), and Meikle et al. (2008)
	JAK/ STAT	*In vivo:* pilocarpine-induced status epilepticus (rat); kainic acid-induced epilepsy (rat).	Mainly pathogenic. STAT3 activation induces neuroinflammation, reactive gliosis, and epileptogenesis following seizures. SOE: High.	Gautam et al. (2021), Grabenstatter et al. (2014), Raible et al. (2014), Lund et al. (2008), Xu et al. (2011), and Choi et al. (2003b)
TBI	RAS/ ERK	*In vivo:* controlled cortical impact (CCI) or controlled mechanical cortical injury (mouse, rat). *In vitro:* primary neuron–astrocyte co-cultures (rat).	Mainly pathogenic. Sustained activation exacerbates oxidative damage, cerebral edema, neuroinflammation, neuronal apoptosis. Transient ERK activation may contribute to neuronal survival and repair. SOE: high.	Wang et al. (2025b), Zhang C. et al. (2024), Mori et al. (2002), amnd Zheng Y. et al. (2022)
	PI3K/ AKT	*In vivo:* controlled cortical impact (CCI)/controlled mechanical cortical injury (mouse, rat). *In vitro:* LPS-stimulated primary microglia (rat).	Mainly protective. Reduces neuronal apoptosis, attenuates neuroinflammation, and improves cognitive and sensorimotor recovery. These effects are mediated in part through downstream mTOR and NRF2 signaling. Excessive or prolonged activation may contribute to maladaptive responses. SOE: high.	Zhu et al. (2026), Zhang et al. (2023), Yang et al. (2024), and Zhang J. et al. (2024)
	JAK/ STAT	*In vivo:* fluid-percussion injury (rat); controlled cortical impact/controlled mechanical cortical injury (mouse, rat). *In vitro:* LPS- or IL-4-stimulated bone marrow-derived macrophages (mouse). Human patients’ brain tissue and serum.	Context-dependent. STAT3 signaling promotes reactive gliosis, neuroinflammation, and ferroptosis during secondary injury, but also mediates neuroprotective, anti-inflammatory, and tissue repair responses. SOE: Moderate/High.	Zhu et al. (2026), Oliva et al. (2012), Youn et al. (2026), Raible et al. (2014), Zhao et al. (2011), Chen et al. (2025), and Barrett et al. (2017)
SAH	RAS/ ERK	*In vivo:* endovascular perforation (mouse); prechiasmatic cistern autologous blood injection (rat); two-hemorrhage model (rabbit)	Mainly pathogenic. Sustained activation promotes neuroinflammation, cerebral vasospasm, BBB disruption, vascular smooth muscle receptor upregulation, neuronal injury, and worsened neurological outcomes. SOE: High.	Khey et al. (2020), Fujimoto et al. (2018), Ansar and Edvinsson (2008), Vikman et al. (2007), Maddahi et al. (2011), Maddahi et al. (2012), Zhang J. et al. (2015), and Chen D. et al. (2009)
	PI3K/ AKT	In vivo: cisterna magna blood injection (rat); endovascular perforation (mouse, rat).	Mainly protective. Preserves BBB integrity, suppresses neuronal apoptosis, reduces neuroinflammation and oxidative stress, and improves neurological outcomes. SOE: Moderate.	Zhuang et al. (2011), Dienel et al. (2021), and Zhu et al. (2018)
	JAK/ STAT	*In vivo:* cisterna magna blood injection (rat); endovascular perforation (mouse)	Context-dependent (dual role). STAT1 is predominantly pathogenic, promoting cerebral vasospasm, neuroinflammation, and cell death. STAT3 has variable roles. Promotes inflammation and pyroptosis, but also mediates neuroprotective, anti-apoptotic, and reparative responses. SOE: Moderate.	Khey et al. (2020), Osuka et al. (2006), Samraj et al. (2014), Osuka et al. (2010), Zheng Z. V. et al. (2022), and Tang et al. (2024)
Ischemic stroke	RAS/ ERK	*In vivo:* MCAO (mouse, rat); four-vessel occlusion (rat)	Context-dependent (dual role). Sustained activation contributes to BBB disruption, neuroinflammation, neuronal apoptosis, and worse neurological outcomes during acute ischemic injury, whereas transient or cell-specific ERK activation can promote neuronal survival, neuroplasticity, and recovery. SOE: High.	Yin et al. (2026), Yan et al. (2007), Wang et al. (2003), Cao et al. (2011), Schanbacher et al. (2022), and Liu et al. (2018)
	PI3K/ AKT	*In vivo:* ischemia/reperfusion and MCAO (rat, mouse). *In vitro:* OGD-treated primary neurons and LPS-treated primary astrocytes (mouse)	Mainly protective. Activation promotes neuronal survival and functional recovery by inhibiting apoptosis, preserving BBB integrity, reducing oxidative stress and neuroinflammation, and enhancing cell survival signaling. SOE: High.	Liu et al. (2025), Amini-Khoei et al. (2019), An et al. (2017), Chen et al. (2012), Chen et al. (2014), Chen and Li (2021), and Dong et al. (2021)
	JAK/ STAT	*In vivo:* ischemia/reperfusion, four-vessel occlusion, and MCAO (rat, mouse). *In vitro:* OGD-treated astrocytes alone or co-cultured with neurons (mouse). Human patient serum	Mainly pathogenic. STAT3 promotes neuroinflammation. SOE: Moderate.	Liang et al. (2016), Li and Zhang (2003), Yu et al. (2013), Choi et al. (2003a), Lei et al. (2011), Borbor et al. (2023), and Adly Sadik et al. (2021)
Spinal Cord Injury	RAS/ ERK	*In vivo:* quisqualic acid intraspinal injection, transection and compression (rat); contusion (mouse)	Dual role. Hyperphosphorylated in neurons and astrocytes. Induces pathogenic proinflammatory cytokines and apoptosis. Neuronal ERK signaling may contribute to neurite outgrowth. SOE: moderate.	Lin et al. (2025), Yu and Yezierski (2005), Li J. et al. (2021), Xu et al. (2006), and Zhao et al. (2019)
	PI3K/ AKT	*In vivo:* compression, contusion, weight-drop and transection (rat); SCI (mouse). *In vitro:* LPS-treated human neuroblastoma cell line SH-SY5Y	Predominantly protective. Upregulated post-SCI. Promotes neuronal survival, axonal growth, remyelination, angiogenesis, and functional recovery while suppressing apoptosis and mediating inflammation. mTOR stimulation in neurons promote axon regrowth. SOE: high.	Xiao et al. (2022), Tang et al. (2019), Lv X. et al. (2024), Wei et al. (2021), Zhang P. et al. (2015), Liu et al. (2010), Jung et al. (2014), and Chen G. et al. (2020)
	JAK/ STAT	*In vivo:* crush, compression and weight-drop (mouse). *In vitro:* model with primary astrocytes and cocultures with meningeal fibroblasts and macrophages, and primary microglia (mouse).	Mainly pathogenic. Cytokine-induced in astrocytes. Upregulates GFAP, inflammatory mediators and scar-related genes. Promotes astrocyte survival. SOE: high.	Herrmann et al. (2008), Wanner et al. (2013), Ageeva et al. (2024), Chen et al. (2026), and Yamauchi et al. (2006)
Olfactory Injury	RAS/ ERK	*In vivo:* Triton X-100-induced OE lesion (zebrafish); OE study (mouse). *In vitro:* model with primary OE (mouse).	Protective. Activated. Induces progenitor-cell proliferation and differentiation, restoring olfactory neurons. SOE: moderate.	Sireci et al. (2024), Jia and Hegg (2012), and Chen et al. (2017)

AKT, protein kinase B (PKB); BBB, blood–brain barrier; BMDM, bone marrow-derived macrophages; CSP, chondroitin sulfate proteoglycan; ERK, extracellular signal-regulated kinase; FCD, focal cortical dysplasia; GFAP, glial fibrillary acidic protein; IL, interleukin; IOP, intraocular pressure; JAK, janus kinase; LPS, lipopolysaccharide; MCAO, *Middle cerebral artery occlusion*; mTOR, mammalian target of rapamycin; NRF2, nuclear factor erythroid 2-related factor 2; OE, olfactory epithelium; OGD, oxygen–glucose deprivation; ON, optic nerve; PI3K, phosphoinositide 3-kinases; RAS, rat sarcoma vírus; RGC, retinal ganglion cells; SAH, subarachnoid hemorrhage; SCI, spinal cord injury; SOE, Strength of evidence; STAT, signal transducer and activator of transcription; TSC, Tuberous sclerosis complex.

**Table 2:** EGFR downstream pathways in neurodegenerative diseases.

Disease	Pathway	Model	Role	**Citation**
PD	RAS/ ERK	*In vitro:* 6-OHDA -treated B65 cells (rat); MPP-treated SH-SY5Y cells (human). Analysis of human patients’ brain	Predominantly pathogenic associated to chronic activated by stress. Promotes inflammation, oxidative stress, and contributes to α-synuclein toxicity. SOE: moderate	Shalabi et al. (2026), Kulich and Chu (2001), Dzamko et al. (2014), Gómez-Santos et al. (2002), Zhu et al. (2002), and Keshri and Singh (2024)
	PI3K/ AKT	*In vivo:* MPTP injection (mouse); Midbrain 6-OHDA injection (rat). *In vitro:* LPS-treated PC12 cells (rat) and MPP-treated SH-SY5Y cells (human).	Generally protective. Preservs dopaminergic neurons. Excessive mTORC1 impair autophagy and α-synuclein clearance. Excessive PI3K/AKT/GSK-3β is detrimental. SOE: moderate	Dong-Chen et al. (2023), Ji et al. (2019), Yang et al. (2018), Zhu et al. (2012), Huang et al. (2019), Goyal et al. (2023), AlRuwaili et al. (2025), Khan et al. (2023), and Keshri and Singh (2024)
	JAK/ STAT	*In vivo:* 61 α-Syn transgenic and MPTP injection (mouse); 6-OHDA striatum injection (rat). *In vitro:* 6-OHDA-treated N27 cells (rat) and BV-2 cells (mouse)	Dual role. STAT3 activation in microglia and astrocytes promotes neuroinflammation and dopaminergic neurodegeneration. Neuronal STAT3 activation/overexpression supports neuronal survival, mitochondrial function, and improves motor deficits. SOE: low	Hong et al. (2024), Jain et al. (2021), Smit et al. (2024), and Wang et al. (2016)
AD	RAS/ ERK	*In vivo:* APP CRISPRi and Aβ hippocampal injection (mouse). *In vitro:* APP-null B103 cells and primary neurons (rat&mouse) and ESC and iPSC derived human neurons, primary glia and MEFs (mouse). Analysis of human patients’ brain.	Mainly pathogenic. Aβ oligomers and cellular stress induce sustained Ras/ERK activation, which promotes tau and APP phosphorylation, neuroinflammation, synaptic dysfunction, neuronal stress responses, and neurodegeneration. SOE: moderate	Kirouac et al. (2017), Khezri et al. (2023), Huang et al. (2017), Faucher et al. (2015), and Russo et al. (2002)
	PI3K/ AKT	*In vivo:* APP/PS1 and SAMp8 transgenic mouse; human Aβ42 expressed in Drosophila. *In vitro:* primary neurons and N2a cells (rat&mouse). Analysis of human patients’ brain.	Mainly protective. Inhibits GSK3β and preserves neuronal survival. Aβ may interrupt the PI3K-Akt–mTOR and may inhibit PI3K/AKT/GSK-3β causing neuronal death and tau hyperphosphorylation. SOE: high	Bhatia et al. (2025), Chen T. J. et al. (2009), Jimenez et al. (2011), Arnés et al. (2020), Kitagishi et al. (2014), Gabbouj et al. (2019), and Bian et al. (2021)
	JAK/ STAT	*In vivo:* APP/PS1 and Tg2576 (mouse). *In vitro:* F11 cells (mouse).	Dual role (mainly pathogenic). STAT3 induces astrocyte and microglia reactivity and neuroinflammation, but may support Aβ clearance. Aβ-dependent neuronal STAT3 inactivation causes memory loss through cholinergic dysfunction. SOE: moderate	Rusek et al. (2023), Chiba et al. (2009a), Hashimoto et al. (2005), Chiba et al. (2009b), Ben Haim et al. (2015), Jain et al. (2021), and Wen and Hu (2024)
ALS	RAS/ ERK	*In vivo:* SOD1(G93A) transgenic mouse, Drosophila model, human study; *In vitro:* spinal cords (rat), Neuro-2A and primary cells (mouse), and SH-SY5Y and patient-derived iPSCs (human). Analysis of human patients’ spinal cord.	Pathogenic. Sustained activation under pathological stress conditions. Contributes to inflammation, excitotoxicity, and motor neuron degeneration. SOE: moderate	Sahana and Zhang (2021), Ayala et al. (2011), Chung et al. (2005), Apolloni et al. (2013), Xia et al. (2015), Yue et al. (2023), Magrì et al. (2023), Sahu et al. (2021), and Bhinge et al. (2017)
	PI3K/ AKT	*In vivo:* SOD1(G93A) transgenic mouse. Human patients’ study.	Protective. Preserves motor neuron survival. SOE: moderate	Nogueira-Machado et al. (2025), Qi et al. (2022), Peviani et al. (2007), Gotkine et al., 2025, and Zheng et al. (2023)
	JAK/ STAT	*In vivo:* SOD1(G93A) transgenic mouse and methylmercury-exposed rat. Analysis of human patients’ nervous tissues.	Pathogenic. STAT3 activation induces astrocyte reactivity and neuroinflammation, and muscle atrophy. SOE: moderate	Richardson et al. (2023), Ohgomori et al. (2017), Shibata et al. (2010), Tortarolo et al. (2024), Founta et al. (2023), Kumar et al. (2024), and Apolloni and D'Ambrosi (2025)
MS	RAS/ ERK	*In vivo:* cuprizone or lysolecithin-induced demyelination and EAE (mouse). *In vitro:* primary mouse cells and patients’ derived PBMCs (human).	Pathogenic. Enhanced in activated microglia, astrocytes. Increases pro-inflammatory neurotoxic, and extracellular matrix mediators release, impairing oligodendrocyte survival and remyelination. SOE: high	El-Dessouki et al. (2026), Naserpour et al. (2025), Ten Bosch et al. (2021), Okazaki et al. (2016), Kotelnikova et al. (2019), Michel et al. (2015), Birkner et al. (2017), and Brereton et al. (2009)
	PI3K/ AKT	*In vivo:* EAE (mouse&rat). *In vitro:* models with primary mouse cells.	Mainly protective. Promotes oligodendrocyte survival and remyelination. However, it may promote pathogenic neuroinflammation. SOE: moderate	El-Dessouki et al. (2026), Naserpour et al. (2025), Kumar et al. (2013), Salem et al. (2025), Mammana et al. (2018), and Wu et al. (2025)
	JAK/ STAT	*In vivo:* EAE (mouse). *In vitro:* primary mouse cells and patients’ derived PBMCs (human). Analysis of human patients’ blood.	Pathogenic. STAT3 drives demyelination by promoting pathological neuroinflammation. Induces Th1 and Th17 differentiation and inflammatory monocytes infiltration. SOE: high	El-Dessouki et al. (2026), Naserpour et al. (2025), Kotelnikova et al. (2019), Liu et al. (2014), Shao et al. (2023), Szewczak et al. (2025), and Dang et al. (2021)
HD	RAS/ ERK	*In vivo:* R6/2 mouse, Drosophila and 3-nitropropionic acid-induced (rat) models. *In vitro:* PC12 cells (rat), striatal knock-in cell lines (mouse) and patient-derived iPSCs (human)	Protective. ERK activation promotes neuronal survival. mHtt impairs ERK-dependent BDNF signaling and glutamate transporter expression, increasing excitotoxicity. SOE: moderate	Jurcau (2022), Krzystek et al. (2025), Maher et al. (2011), Sarantos et al. (2012), El-Shamarka et al. (2022), Bodai and Marsh (2012), and Yusuf et al. (2021)
	PI3K/ AKT	*In vivo:* transgenic and quinolinic acid-induced mouse models; 3-nitropropionic acid-induced (rat). *In vitro:* striatal primary and NPC lines (mouse) and primary cells and cell lines (human). Analysis of human patients’ brain.	Mainly protective. Promotes neuronal survival. AKT phosphorylation of mHTT inhibits mHTT-induced apoptosis. Downstream mTOR blocks autophagy. Inhibiting mTOR clears mHTT aggregates. Disrupted in HD. SOE: moderate	Elgindy et al. (2026), Sayed et al. (2020), Saavedra et al. (2010), Humbert et al. (2002), Ribeiro et al. (2014), and Colin et al. (2005)
	JAK/ STAT	*In vivo* Hdh140 transgenic and lentiviral vector-based models (mouse&monkey); 3-nitropropionic acid-induced (rat).	Protective. STAT3 activation induces beneficial astrocyte reactivity. Blocking STAT3 increasesmHTT aggregates. SOE: moderate	Abjean et al. (2023), Ben Haim et al. (2015), and Ibiayo et al. (2025)

6-OHDA, 6-hydroxydopamine; AD, Alzheimer’s disease; AKT, protein kinase B (PKB); ALS, Amyotrophic lateral sclerosis; APP, amyloid precursor *protein*; ATP, adenosine triphosphate; BDNF, *brain-derived neurotrophic factor*; EAE experimental allergic encephalomyelitis; ERK, extracellular signal-regulated kinase; ESC, embryonic stem cell; GSK, glycogen synthase kinase; HD, Huntington’s disease; iPSCs, induced pluripotent stem cells; JAK, janus kinase; murine embryonic fibroblasts; MEF, murine embryonic fibroblasts; MPP, 1-methyl-4-phenylpyridinium iodide; MPTP, 1-methyl-4-phenyl-1,2,3,6-tetrahydropyridine; MS, Multiple sclerosis; mTOR, mammalian target of rapamycin; mHTT, mutant huntingtin; NPC; neuronal progenitor cell; PBMCs, peripheral blood mononuclear cells; PD, Parkinson’s disease. PI3K, phosphoinositide 3-kinases; PTEN, phosphatase and tensin homolog; RAS, rat sarcoma virus; RPE, retinal pigment epithelial; SOE, Strength of evidence; STAT, signal transducer and activator of transcription.

EGFR is expressed in both the central and peripheral nervous systems, particularly during development, and EGFR-deficient mice exhibit multiple neural abnormalities (Sibilia et al., 1998; Kornblum et al., 1998). In the adult brain, EGFR signaling plays important roles in neural stem and progenitor cell proliferation, neurogenesis, neurite outgrowth, astrocyte activation, and remyelination. EGF and TGF-*α* promote neural progenitor proliferation and neuronal survival, while HB-EGF and BTC enhance neurogenesis and neural precursor survival (Doetsch et al., 2002; Bath and Lee, 2010; Getchell et al., 2000). EGFR signaling in neural stem cell niches regulates progenitor expansion and differentiation, although prolonged activation may skew differentiation toward astrocytic or oligodendroglial lineages at the expense of neurons (Kuhn et al., 1997; Gonzalez-Perez et al., 2009). These findings suggest that tightly regulated EGFR signaling is required to maintain a balance between stem cell renewal and lineage commitment within the adult nervous system. Notably, the downstream biological response is influenced not only by receptor activation itself but also by the specific ligand involved, the composition of ErbB receptor dimers, and the intracellular signaling pathways preferentially engaged.

In neurons and astrocytes, EGFR signaling can promote neurite extension, neuronal survival, and the formation of a pro-regenerative environment, largely through AKT and ERK signaling pathways (Goldshmit et al., 2004a; Goldshmit et al., 2004b; Liu and Neufeld, 2004). However, sustained EGFR activation may also promote reactive astrogliosis, neuroinflammation, and glial scar formation, highlighting the context-dependent nature of EGFR signaling in the nervous system (Liu et al., 2006; Romano and Bucci, 2020; Badenes, 2024; Qu et al., 2012). A growing body of evidence indicates that transient EGFR activation following injury often promotes regenerative processes, whereas chronic or excessive activation, particularly in reactive glial populations, may drive inflammatory and neurodegenerative responses. This apparent functional dichotomy may explain why both EGFR agonists and inhibitors have shown beneficial effects in different experimental models of neurological disease. Importantly, these seemingly opposing findings should not be viewed as contradictory but rather as reflecting the highly dynamic and spatiotemporally regulated nature of EGFR signaling within the injured or diseased nervous system. In demyelinating conditions, reactivation of EGFR signaling in oligodendrocyte lineage cells enhances oligodendrogenesis and remyelination (Aguirre et al. (2007); Scafidi et al., 2014; Bartus et al., 2019). Overall, EGFR signaling exerts complex and highly cell-type-dependent effects in the nervous system, contributing to both regenerative and pathological processes depending on the cellular and disease context. Understanding the factors that determine this switch between beneficial and detrimental EGFR signaling remains a major challenge and is central to the development of EGFR-targeted therapies for neurological disorders. Throughout this review, we examine the evidence supporting this context-dependent model and discuss how a better understanding of EGFR signaling dynamics may guide the development of more precise therapeutic strategies for neurodegenerative diseases.

### 1.2 EGFR in neural repair

EGFR signaling is rapidly activated following central nervous system (CNS) injury in glial cells and neural progenitors, where it contributes to tissue repair, neurogenesis, and cell survival (Liu et al., 2006; Romano and Bucci, 2020, Li Z.W. et al., 2021). However, EGFR signaling exerts highly context-dependent effects. Moderate and transient activation of EGFR can promote regeneration by enhancing neural progenitor proliferation, neuroblast migration, neurite outgrowth, and remyelination, whereas sustained or excessive activation often drives reactive astrogliosis, neuroinflammation, glial scar formation, and neurodegeneration (Liu and Neufeld, 2003; Qu et al., 2012; Li et al., 2014). These contrasting effects likely reflect differences in the timing, intensity, cellular localization, and duration of EGFR signaling following injury. In particular, ligand-driven activation of EGFR in neural progenitors and oligodendrocyte lineage cells generally promotes regenerative responses, whereas prolonged activation in reactive astrocytes and inflammatory cells may exacerbate tissue damage and impede recovery. These context-dependent effects are also likely influenced by selective engagement of downstream signaling pathways, with PI3K/AKT and ERK signaling generally associated with cell survival and regeneration, whereas sustained activation of inflammatory signaling pathways, including JAK/STAT, may contribute to reactive gliosis and chronic neuroinflammation. Consistent with this dual role, both EGFR activation and inhibition have shown beneficial effects across different injury models, depending on timing, cell type, and signaling context. Key findings are summarized in Table 3.

**Table 3:** EGFR in neural repair.

Condition	Model	Results	Citation
Optic nerve injury	*In vivo:* transient eye ischemia; ON transection (rat); ON crush model (mouse). *In vitro:* CGNs and DRG neuron and retinal explants plus myelin (rat). Neuronal tissue analysis (human).	EGFR overactivation in astrocytes leads to reactive astrocytes, neuronal degeneration. SOE: Moderate.	Liu et al. (2006) and Koprivica et al. (2005)
	*In vivo:* ON crush & sciatic nerve impantation (rat). *In vitro:* retinal explants and primary retinal ganglion cell plus myelin (rat).	EGFR inhibition induces off-target effects, as neurotrophin release, elevated cAMP, p75^NTR^ proteolysis, promoting neurite outgrowth. SOE: Low/moderate.	Douglas et al. (2009) and Berry et al. (2011)
Glaucoma	*In vitro:* primary ON head astrocytes exposed to elevated hydrostatic pressure (human). ON head tissue analysis (human).	EGF promotes astrocyte activation, leading to neurotoxicity and axonal degeneration. SOE: Moderate.	Liu and Neufeld (2003)
	*In vivo:* chronic glaucoma (rat).	EGFR inhibition promotes axon regeneration and retinal ganglion cell protection. SOE: Moderate.	Liu et al. (2006)
	*In vivo:* DBA/2 J model (mouse).	EGFR inhibition worsens disease via reduced C3 release. SOE: Moderate/high.	Harder et al. (2017)
Epileptic seizures	*In vivo:* kainic acid–induced MTLE (mouse). *In vitro:* hippocampal NSCs (mouse).	HB-EGF/EGFR causes NSC reactivity and impaired neurogenesis. SOE: Moderate.	Pastor-Alonso et al. (2024)
Brain injury	*In vivo:* corpus callosum lysolecithin injection-induced focal demyelination (mouse).	EGFR overexpression leads to OPC expansion and remyelination. SOE: Moderate.	Aguirre et al. (2007)
	*In vivo:* middle cerebral artery occlusion (mouse).	EGF leads to reduced neuron loss, increased proliferation (SVZ/hippocampus); expansion of transit-amplifying cells in SVZ. SOE: Moderate/high.	Ninomiya et al. (2006) and Sun et al. (2010)
	*In vitro:* OGD in primary astrocytes and astrocytic cell line (rat).	EGFR overactivation in astrocytes and progenitors. SOE: Moderate.	Chen et al. (2019)
	*In vivo:* controlled mechanical cortical brain injury (mouse).	TGF-α promotes proliferation but inhibits neuroblast maturation/migration. SOE: Moderate.	Gómez-Oliva et al. (2023)
	*In vivo:* controlled mechanical cortical brain injury (mouse).	EGFR inhibition increases neuroblast migration, reduces astrocyte activation. SOE: Moderate.	Gómez-Oliva et al. (2023) and Yu et al. (2024)
	*In vivo:* controlled mechanical cortical brain injury (rat).	Myricetin activates EGFR-AKT, reducing microglial activation. SOE: Low.	Wang et al. (2023)
SAH	*In vivo*: endovascular perforation model (rat, mouse); Intracisternal injection of Tenascin-C (rat). Serum and cerebrospinal fluid from aneurysmal SAH patients (human).	HB-EGF/tenascin-C promotes vasospasm, apoptosis, neuroinflammation. SOE: High.	Shiba et al. (2014), Fujimoto et al. (2016), Suzuki et al. (2020), and Nakano et al. (2023)
	*In vivo:* endovascular perforation model (mouse).	EGFR inhibition leads to reduced vasospasm and apoptosis via ERK/NF-κB suppression. SOE: High.	Nakano et al. (2019) and Nakano et al. (2023)
	*In vivo:* endovascular perforation model (rat).	GPR30 activation leads to neuroprotection via SRC/EGFR/STAT3. SOE: Moderate.	Peng et al. (2019)
Stroke	*In vivo:* middle cerebral artery occlusion (mouse).	EGF leads to proliferation, migration, neurogenesis. SOE: Moderate/high.	Teramoto et al. (2003) and Ninomiya et al. (2006)
Spinal cord injury	*In vivo* weight-drop injury (rat, mouse); Crush and transection injury (mouse); Transection injury (zebrafish). *In vitro:* LPS-treated microglia cells and astrocyte scratch wound models (rat).	EGFR inhibition leads to reduced gliosis, increased myelination, oligodendrocyte protection. SOE: Moderate/ high.	Erschbamer et al. (2007), Qu et al. (2012), Li et al. (2014), Zhang et al. (2016), and Cigliola et al. (2023)
	*In vivo:* moderate contusion injury (mouse). *In vitro:* spinal cord neural progenitor cell and astrocytes (wound healing) and with primary DRG cells (mouse).	EGFR deletion leads to defective glial borders; EGFR needed for supportive astrocytes. SOE: High.	White et al. (2011)
	*In vivo:* weight-drop injury (mouse). *In vitro:* primary OPCs, and NCSs plus myelin (rat).	EGFR inhibition promotes neurogenesis via RAS–ERK/TRIM32. SOE: Moderate/high.	Ju et al. (2012) and Xue et al. (2020)
	*In vivo:* moderate contusion injury (rat). *In vitro:* model with a brain MEC line (human).	EGF preserves barrier via PI3K/AKT/Rac1. SOE: Moderate.	Zheng et al. (2016)
	*In vivo:* transection injury (rat); *In vitro:* primary undifferentiated and differentiated NSCs (rat).	VEGF induces NSC proliferation via EGFR-VEGFR2. SOE: Moderate.	Liu et al. (2019)
	*In vivo:* weight-drop injury (rat). *In vitro:* OGD/ reoxygenation model with primary cells (rat).	HB-EGF/EGFR activation leads to barrier disruption, astrogliosis. SOE: Moderate.	Li Z. W. et al. (2021)
	*In vivo:* moderate contusion and crush injury (mouse); Transection injury (mouse, zebrafish). *In vitro:* primary NSC and cortical neurons (mice).	EGFR activation (exosomes/HB-EGF) promotes recovery. SOE: Moderate/high.	Qin et al. (2024) and Cigliola et al. (2023)
SCI I-R	*In vivo:* abdominal aorta temporally clamp (mouse). *In vitro:* OGD model in primary neurons (mouse).	EGFR overexpression leads to reduced inflammation, pyroptosis, improved barrier. SOE: Moderate.	Lv S. et al. (2024)
OE injury	*In vivo:* analysis of TGF-α-transgenic animals OE (mouse); Methimazole-induced OE lesion and olfactory bulbectomy (mouse); Triton X-100-induced OE lesion (zebrafish). *In vitro:* primary OE organoids (mouse).	EGFR and ligands upregulated; EGFR activation leads to basal cell proliferation and neurogenesis (MAPK/ERK). SOE: Moderate/high.	Getchell et al. (2000), Chen Z. H. et al. (2020), and Sireci et al. (2024)

AKT, protein kinase B (PKB); CGNs, cerebellar granule cells; DRG, dorsal root ganglion; EGF, epidermal growth factor; EGFR, epidermal growth factor receptor; ERK, extracellular signal-regulated kinase; GPR30, *G protein-coupled receptor 30,* HB-EGF, heparin-binding EGF-like growth factor; I-R, ischemia–reperfusion; MAPK, mitogen-activated protein kinase; MEC, microvascular endothelial cell; MTLE, mesial temporal lobe epilepsy; NF-κB, nuclear factor kappa B; NSC, neural stem cell; OE, olfactory epithelium; OGD, oxygen/glucose deprivation; ON, optic nerve; OPC, oligodendrocyte progenitor cell; PI3K, phosphoinositide 3-kinases; RAS, rat sarcoma virus; SAH, subarachnoid hemorrhage; STAT, signal transducer and activator of transcription; SCI, spinal cord injury; SOE, Strength of evidence; SVZ, subventricular zone; TGF-α, transforming growth factor-α; TRIM32, tripartite motif 32; VEGF, vascular endothelial growth factor; VEGFR, vascular endothelial growth factor receptor.

In models of traumatic brain injury and ischemic stroke, EGF promotes neural repair by enhancing progenitor proliferation, neurogenesis, and neuronal survival, particularly within the subventricular zone and hippocampus (Teramoto et al., 2003; Sun et al., 2010). Similarly, EGFR signaling contributes to oligodendrocyte progenitor expansion and remyelination following demyelinating injury (Aguirre et al., 2007). These findings support a role for transient EGFR activation in restoring neural cell populations and promoting functional recovery after injury. Conversely, excessive EGFR activation in reactive astrocytes has been associated with neurotoxicity, impaired neurogenesis, and inflammatory gliosis. Accordingly, pharmacological inhibition of EGFR has been shown to reduce astrocyte and microglial activation, enhance neuroblast migration, promote axonal regeneration, and improve functional recovery in several CNS injury models, including optic nerve injury, spinal cord injury, and subarachnoid hemorrhage (Koprivica et al., 2005; Erschbamer et al., 2007; Gómez-Oliva et al., 2023; Nakano et al., 2023). Collectively, these studies suggest that the therapeutic value of EGFR inhibition may arise from limiting maladaptive glial responses rather than suppressing regenerative EGFR signaling per se. Together, these findings emphasize that the biological outcome of EGFR modulation is determined less by whether the pathway is activated or inhibited, and more by when, where, and in which cell types the intervention occurs.

In spinal cord injury, EGFR signaling has been implicated in both regenerative and pathological processes. EGFR inhibition suppresses astrogliosis and glial scar formation, protects oligodendrocytes from apoptosis, and promotes neurogenesis and myelination (Qu et al. 2012; Li et al., 2014; Zhang et al., 2016). In contrast, some studies suggest that EGFR activation in astrocytes may support neurite outgrowth and tissue repair under specific conditions, while EGFR overexpression or delivery of EGFR-containing exosomes, HB-EGF, or EGF can improve recovery after spinal cord injury (White et al., 2011; Cigliola et al., 2023, Qin et al., 2024). At first glance, these findings appear contradictory. However, they may instead reflect distinct phases of the injury response. Early and controlled EGFR activation may facilitate cell survival, axonal growth, and tissue repair, whereas persistent EGFR activation during later stages may promote chronic gliosis and scar formation. Thus, the timing of intervention may be a critical determinant of whether EGFR activation or inhibition is therapeutically beneficial. Together, these findings suggest that the effects of EGFR signaling in neural repair are highly dependent on the magnitude, duration, and cellular localization of pathway activation. Future studies combining cell-type-specific genetic models with single-cell and spatial transcriptomic approaches will be important for defining the spatiotemporal dynamics of EGFR signaling during neural repair and for identifying therapeutic windows that maximize regeneration while minimizing pathological remodeling. A better understanding of the spatiotemporal regulation of EGFR signaling following CNS injury will be essential for developing therapeutic approaches that selectively enhance regenerative processes while minimizing pathological outcomes.

### 1.3 EGFR in neurodegenerative diseases

**Figure 2:**
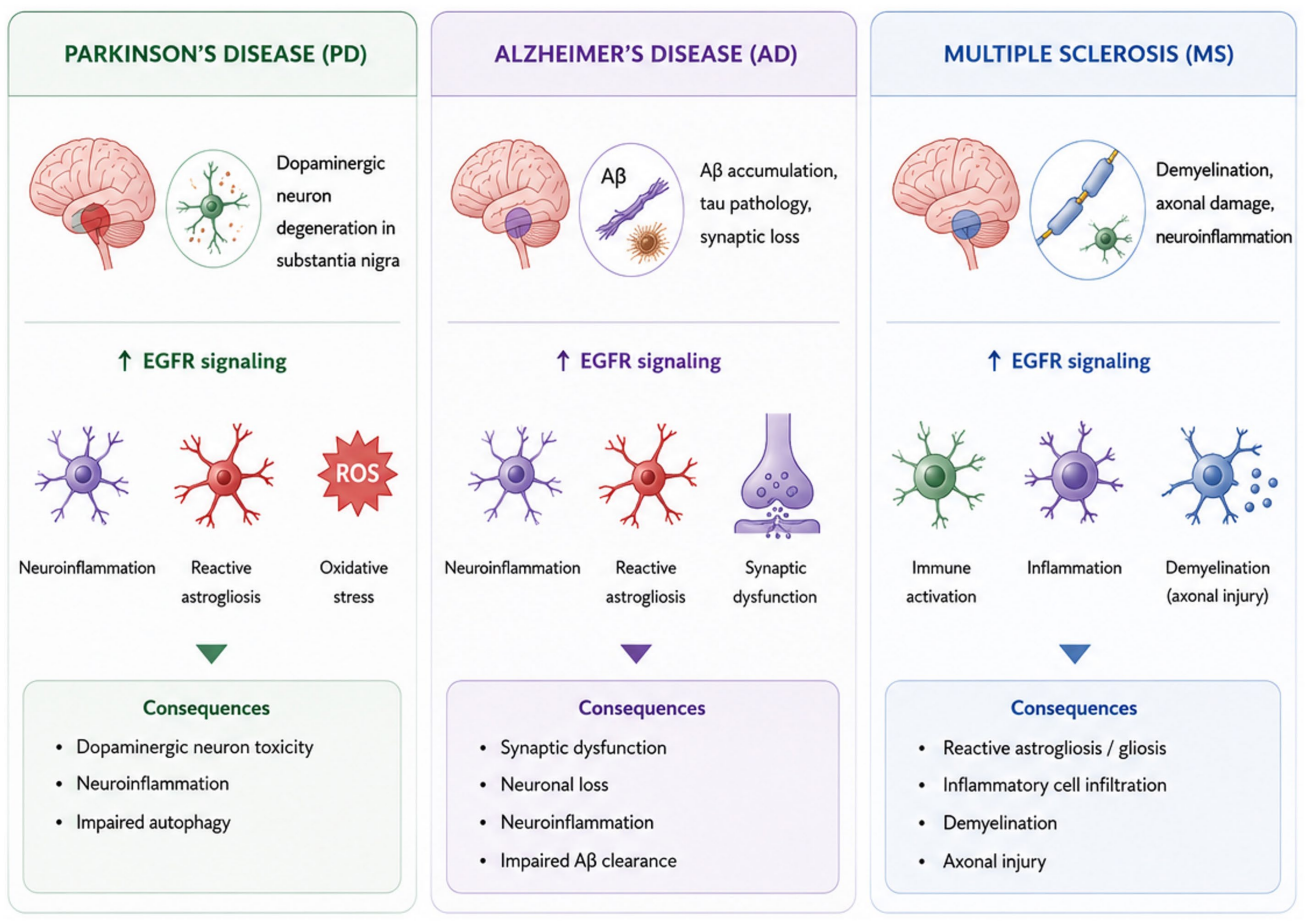
EGFR dysfunction in neurodegenerative diseases. Dysregulated EGFR signaling contributes to the pathogenesis of multiple neurodegenerative disorders through mechanisms involving neuroinflammation, reactive gliosis, oxidative stress, synaptic dysfunction, and neuronal injury. In Parkinson’s disease (PD), increased EGFR signaling is associated with dopaminergic neuron degeneration in the substantia nigra, neuroinflammation, reactive astrogliosis, oxidative stress, and impaired autophagy. In Alzheimer’s disease (AD), EGFR dysregulation is linked to amyloid-β (Aβ) accumulation, tau pathology, synaptic dysfunction, neuronal loss, impaired Aβ clearance, and chronic neuroinflammation. In Multiple sclerosis (MS), altered EGFR signaling contributes to immune activation, inflammatory gliosis, demyelination, axonal injury, and neuroinflammatory responses. The role of EGFR in Amyotrophic lateral sclerosis (ALS) and Huntington’s disease remains poorly understood, and the available evidence is still limited. Collectively, these findings highlight shared EGFR-associated pathological mechanisms across neurodegenerative diseases and support the concept that aberrant EGFR signaling may contribute to disease progression in a context-dependent manner. This figure was generated using BioRender.

Alterations in EGFR expression and signaling have been implicated in multiple neurodegenerative diseases. Key findings are summarized in Table 4 and in Figure 2. However, the current evidence remains limited and, in some cases, conflicting. These inconsistencies likely reflect the complex and context-dependent nature of EGFR signaling, whereby physiological EGFR activity may support neuronal survival and tissue homeostasis, while chronic or dysregulated activation may contribute to neuroinflammation and neurodegeneration. Furthermore, much of the current evidence is derived from animal models and *in vitro* studies, highlighting the need for additional mechanistic and translational studies in humans. Importantly, the available evidence suggests that EGFR does not function as a universal driver of neurodegeneration, but rather as a context-dependent signaling node whose effects vary according to disease stage, cellular environment, and the mechanisms underlying individual neurodegenerative disorders.

**Table 4:** EGFR in neurodegenerative diseases.

Disease	Model	Results	Citation
PD	*In vivo:* intra-nigral 6-OHDA infusion (rat). Analysis of patients’ brains (human).	Decreased EGF and EGFR levels. SOE: Low/moderate.	Iwakura et al. (2005) and Iwakura and Nawa (2013)
	*In vitro:* parkin or Eps15 transfected COS-7 (monkey), HEK293, HeLa (human), HER14 (mouse), MEFs and brain synaptosomes from parkin knockout animals (mouse).	Parkin normally restrains EGFR endocytic trafficking and degradation by ubiquitinating Eps15; Loss of Parkin leads to increased EGFR degradation and reduced AKT signaling. SOE: Moderate.	Fallon et al. (2006) and Husnjak and Dikic (2006)
	*In vitro:* LRRK2 transfected HEK293 and HeLa cells, and primary skin fibroblasts from LRRK2 G2019S mutant patients (human). Analysis of brains from LRRK2 G2019S mutant patients (human).	Mutant LRRK2 impairs Rab7-dependent late endosomal trafficking, delaying EGFR degradation. SOE: Moderate.	Gómez-Suaga et al. (2014)
	*In vivo:* α-synuclein-PFF intracerebral administration (mouse). *In vitro:* α-synuclein-PFFs uptake in the human neuroblastoma SK-N-SH cell line and primary mouse cortical neurons.	EGFR inhibition (AZD3759) reduces α-synuclein pathology. SOE: Moderate.	Tavassoly et al. (2021)
	*In vivo:* rotenone subcutaneously intoxication (rat).	EGFR inhibition (Lapatinib) has antioxidant effects and restores dopaminergic neurons. SOE: Low.	Mansour et al. (2024)
	*In vitro:* human neuroblastoma SK-N-SH cell line treated with rotenone. Analysis of human PD patients’ brain.	EGFR phosphorylates DNAJB1 promoting α-synuclein clearance. SOE: Low/moderate.	Huang et al. (2025)
AD	Analysis of human patients’ brain.	TGF-alpha and EGFR expressed in AD neurons/ neuritic plaques. SOE: Low/moderate.	Birecree et al. (1988) and Ferrer et al. (1996)
	*In vivo:* APP/PS1 transgenic mouse, Aβ42 transgenic Drosophila.	Aβ binds EGFR causing sustained activation. EGFR inhibition rescues Aβ-induced memory loss. In late stage, Aβ induce a reduction of the total EGFR level. SOE: Moderate.	Wang et al. (2012) and Wang et al. (2013)
	*In vivo:* APP/PS1 transgenic mouse, d-galactose induced AD-like rat model; Aβ-overexpressing *Drosophila* model.	Increased EGFR activation promotes Aβ toxicity, and reduced cognitive function. SOE: Moderate.	Prüßing et al. (2013), Özbeyli et al. (2017), Choi et al. (2023), and Jayaswamy et al. (2023)
	*In vivo:* E4FAD transgenic mouse.	EGF prevents cognitive decline, microbleeds and cerebrovascular dysfunction. SOE: Moderate.	Thomas et al. (2016)
	*In vitro:* SH-SY5Y neuroblastoma and HeLa cell lines (human).	APP binds to EGF/HB-EGF. EGF/HB-EGF increase APP levels. APP and EGF/EGFR have a synergistic effect promoting ERK signaling and neuritogenesis. SOE: Low/moderate.	da Rocha et al. (2021)
ALS	Human patients CSF.	Reduced EGF levels. SOE: Low.	Cieślak et al. (1986)
	*In vivo:* SOD1-G93A transgenic mouse. Human patients’ spinal cord.	Increased EGFR mRNA expression in parallel with the deterioration of motor functions. SOE: Low/moderate.	Offen et al. (2009)
	*In vivo:* SOD1-G93A transgenic mouse	EGFR inhibition (erlotinib) delays onset but no survival benefit. SOE: Moderate.	Le Pichon et al. (2013)
MS	*In vivo:* lysolecithin-induced corpus callosum demyelination (mouse)	EGF/HB-EGF increase SVZ cell proliferation, mobilization, derived oligodendrocytes, and differentiation toward astrocytes (HB-EGF) or oligodendrocytes (EGF). SOE: Moderate.	Cantarella et al. (2008) and Gonzalez-Perez et al. (2009)
	*In vivo:* Theiler’s virus-induced demyelination (mouse)	EGF associated with repair. SOE: Moderate.	Bieber et al. (2010)
	*In vivo:* cuprizone-induced demyelination (mouse)	EGF mRNA is upregulated during remyelination. SOE: Low/moderate.	Gudi et al. (2011)
	*In vivo:* MOG-induced EAE (mouse) and xenogeneic spinal cord homogenate-induced (rat)	EGF + GHRP-6 ameliorates EAE and survival by reducing neuroinflammation, microvascular damage, oxidative stress, and increasing brain IGF-1. SOE: Low/Moderate.	del Barco et al. (2011)
	*In vivo:* MOG-induced EAE and cuprizone-induced demyelination (mouse). Analysis of human patients’ brain	EGFR level is reduced in human chronic lesions. EGF prevents demyelination and clinical signs. SOE: Moderate.	Nicoletti et al. (2019)
	Human patients’ circulating plasma	Low EGF correlates with disease severity; EGF increases with recovery or improved clinical status. SOE: Low.	Tejera-Alhambra et al. (2015) and Reginald McDaniel et al. (2020)
HD	*In vitro:* StHdh ^Q111^ cells and primary striatal cells derived from Hdh^Q111^ mice.	Increased EGF/EGFR downstream signaling (AKT and MEK) alters huntingtin localization, affecting transcription. SOE: Low/moderate.	Bowles et al. (2015)
	*In vitro:* primary fibroblasts from human patients.	mHTT disrupts EGFR trafficking and degradation, leading to altered ERK signaling and reduced EGF-dependent proliferation and migration. SOE: Moderate.	Melone et al. (2013)

Aβ, amyloid-beta; AD, Alzheimer’s disease; AKT, protein kinase B (PKB); MEK, kinases-mitogen-activated protein kinase kinase; ALS, Amyotrophic lateral sclerosis; CNS, Central nervous system; CSF, cerebrospinal fluid; EAE experimental allergic encephalomyelitis; EGF, epidermal growth factor; EGFR, epidermal growth factor receptor; EV, empty vector; GHRP-6, growth hormone releasing peptide-6; HB-EGF, heparin-binding EGF-like growth factor; IGF-1, insulin like growth factor-1; HD, Huntington’s disease; LRRK2, leucine-rich repeat kinase 2; mHTT, mutant huntingtin; MOG, *myelin oligodendrocyte glycoprotein*; MS, multiple sclerosis; PFF, pre-formed fibrils; PD, Parkinson’s disease; 6-OHDA, 6-hydroxydopamine; SVZ, subventricular zone.

#### 1.3.1 Parkinson’s disease

In Parkinson’s disease (PD), patients’ brains show decreased EGF and EGFR levels (Iwakura and Nawa, 2013; Iwakura et al., 2005). Genetic PD models implicate EGFR trafficking: Parkin is mutated in an autosomal recessive form of PD, and its loss promotes EGFR internalization and degradation, reducing EGFR/AKT signaling in neurons (Husnjak and Dikic, 2006; Fallon et al., 2006); Leucine-rich repeat kinase 2 (LRRK2) mutations in PD similarly perturb EGFR recycling (Gómez-Suaga et al., 2014). These deficits in EGFR/AKT may contribute to dopaminergic neuron loss and PD progression, or can also be a consequence of dopaminergic neuron loss due to gene mutations or by other factors. Notably, EGFR inhibition with the tyrosine kinase inhibitor AZD3759 or lapatinib shows beneficial effect in rodents (Tavassoly et al., 2021; Mansour et al., 2024). AZD3759 reduces phosphorylated *α*-synuclein pathology, indicating that EGFR may facilitate pathological protein spread (Tavassoly et al., 2021). Lapatinib exerts antioxidant actions and restores dopaminergic neurons, improving motor impairment and histopathological abnormalities. This dopaminergic effect is mediated by the upregulation of dopamine D3 receptors, increased tyrosine hydroxylase expression, and elevated dopamine levels (Mansour et al., 2024). Importantly, the study by Mansour and colleagues utilized an acute toxin-induced model rather than a synucleinopathy model, and therefore may not fully capture the pathogenic mechanisms underlying human PD. Conversely, some data suggest EGFR activity is neuroprotective, as EGFR-mediated phosphorylation of chaperone DNAJB1 helps clear α-synuclein in Parkinson’s disease (Huang et al., 2025). Taken together, these findings suggest that physiological EGFR signaling may support neuronal maintenance and proteostatic homeostasis, whereas aberrant or sustained EGFR activation may contribute to pathological α-synuclein accumulation and neuroinflammation. This framework may help explain why both impaired EGFR signaling and EGFR inhibition have been reported to produce beneficial effects in distinct PD models, potentially reflecting context-dependent roles of EGFR signaling in disease progression. Whether these apparently opposing roles reflect differences in disease stage, cellular targets, or selective activation of downstream signaling pathways remains an important question for future investigation.

#### 1.3.2 Alzheimer’s disease

In Alzheimer’s disease (AD), several studies report increased EGFR expression and activation in the brain (elevated EGFR phosphorylation) in amyloid precursor protein (APP)/ Presenilin 1 (PS1) mouse models, associated to increased amyloid beta (Aβ) neurotoxicity and possibly neuroinflammation by promoting astrocyte activation (Choi et al., 2023; Jayaswamy et al., 2023; Mansour et al., 2022; Ferrer et al., 1996; Birecree et al., 1988; Özbeyli et al., 2017). The EGFR ligands EGF and HB-EGF upregulate APP protein levels in neuronal cells, and interact with APP to boost ERK signaling and neurite outgrowth which is blocked by EGFR inhibition (da Rocha et al., 2021). Aβ binds to EGFR (Wang et al., 2012) and Aβ deposition elevates EGFR expression and induces sustained activation of EGFR and downstream signaling pathways (Jayaswamy et al., 2023; Wang et al., 2013; Kirouac et al., 2017). EGFR overexpression worsens memory deficits (Prüßing et al., 2013), while EGFR inhibitors ameliorate memory loss (Özbeyli et al., 2017; Wang et al., 2012). However, findings from Drosophila models should be interpreted cautiously, as species-specific differences in EGFR signaling and AD-related pathways may limit their direct translational relevance to human disease. Furthermore, EGFR inhibition may promote autophagy to clear aggregation of toxic amyloid structures (Tavassoly et al., 2020). Collectively, these studies support the notion that chronic EGFR activation contributes to AD pathology by promoting Aβ-mediated toxicity.

However, paradoxically, chronic EGF administration also prevents cognitive dysfunction in a mouse model (possibly by enhancing neurogenesis), despite it does not alter the brain levels of EGFR or Aβ (Thomas et al., 2016). This apparent discrepancy may reflect differences between ligand-mediated physiological EGFR activation and disease-associated receptor activation induced by Aβ accumulation. Whereas pathological EGFR activation appears to exacerbate neurodegeneration, controlled stimulation by endogenous ligands may promote neurogenesis, neuronal survival, or synaptic plasticity. Thus, the biological consequences of EGFR signaling in AD likely depend on both the source and duration of pathway activation. These observations further suggest that selectively modulating pathological EGFR activation while preserving its physiological neurotrophic functions may represent a more effective therapeutic strategy than indiscriminate inhibition of the pathway.

### 1.3.3 Amyotrophic lateral sclerosis

EGFR signaling may also affect amyotrophic lateral sclerosis (ALS) pathology, but data are limited. In ALS, EGF levels are reduced in the cerebrospinal fluid (Cieślak et al., 1986), but EGFR mRNA expression is elevated in spinal cords (Offen et al., 2009), while protein levels are unclear. EGFR inhibitor erlotinib in SOD1-G93A mice shows modest benefits, leading to delayed symptoms-onset, but not extending survival or rescuing neuromuscular synapses (Le Pichon et al., 2013). Overall, the currently available data are insufficient to establish a clear role for EGFR signaling in ALS pathogenesis, highlighting an important area for future investigation. Future studies should determine whether altered EGFR signaling contributes directly to motor neuron degeneration or represents a secondary response to ongoing neurodegeneration.

#### 1.3.4 Multiple sclerosis

Several studies highlight EGFR pathway in remyelination in Multiple sclerosis (MS)-like models. In chemical-induced CNS demyelination model exogenous HB-EGF and EGF promote SVZ proliferation and mobilization and an increase in oligodendrocyte number in the lesion site (Gonzalez-Perez et al., 2009; Cantarella et al., 2008). In encephalomyelitis virus-induced CNS demyelination model high CNS EGF gene expression promotes repair (Bieber et al., 2010), while in myelin ODC glycoprotein (MOG)-induced experimental allergic encephalomyelitis (EAE), chronic treatment with EGF had a preventive effect leading to almost complete absence of signs of demyelination (Nicoletti et al., 2019). In another study, EGF co-administration with growth hormone releasing peptide-6, but not alone, improved the clinical score and survival rate of mild and severe EAE forms (Del Barco et al., 2011). Patients with MS have significantly lower EGF levels in the cerebrospinal fluid (Scalabrino et al., 2010) and in the spinal cord (Scalabrino et al., 2015), but no differences in the mRNA level in cortical lesions neither in chronic or chronic active plaques (Nicoletti et al., 2019). However, they have less EGFR gene expression in chronic active plaques (Nicoletti et al., 2019). Low plasma EGF levels are significantly reduced in the patients with progressive disease compared with those with relapsing remitting forms and to healthy controls. EGF plasma levels are inversely correlated with clinical disability, and low EGF plasma levels combined with other biomarkers (HGF, Eotaxin/CCL11, and MIP-1β/CCL4) predicts the form of progressive MS (Tejera-Alhambra et al., 2015). Blood EGF levels increase after improvement in MS patient’s nutritional and clinical status (Reginald McDaniel et al., 2020). Furthermore, HB-EGF expression is increased in reactive astrocytes of MS brain active lesions (Schenk et al., 2013). Together, these observations suggest that EGFR signaling may have a predominantly regenerative role in MS through its effects on oligodendrocyte biology and remyelination, although local activation of the pathway within inflammatory lesions may exert distinct effects that require further investigation. These findings distinguish MS from other neurodegenerative disorders discussed in this review, as the predominant therapeutic goal may be to enhance regenerative EGFR signaling rather than suppress receptor activity.

#### 1.3.5 Huntington’s disease

EGFR’s role in Huntington’s disease (HD) is not well defined; in HD *in vitro* models, cells carrying mutant huntingtin have increased activation of AKT and MEK signaling activation downstream of the EGFR upon EGF stimulation. This may promote to aberrant huntingtin subcellular localization, altering gene expression (Bowles et al., 2015). In addition, in human HD fibroblasts, mutant huntingtin causes a delay in EGFR degradation and altered trafficking (Melone et al., 2013). Although these findings suggest that mutant huntingtin can influence EGFR signaling dynamics, evidence directly linking EGFR dysfunction to HD pathogenesis remains sparse. Consequently, no clear conclusions can currently be drawn regarding the therapeutic potential of targeting EGFR in HD. Whether this reflects a genuinely limited contribution of EGFR signaling to HD pathogenesis or simply an underexplored area of research remains unclear.

### 1.4 Targeting the EGFR pathway to treat neurodegenerative diseases

Given its dual roles, EGFR represents a challenging but promising therapeutic target. A major obstacle is that EGFR signaling can exert either beneficial or detrimental effects depending on the signaling intensity, ligand availability, receptor dimerization, and downstream pathway bias, the disease context, cell type, and stage of disease progression. Consequently, therapeutic approaches targeting EGFR signaling must consider the temporal and spatial context of pathway modulation, as well as the degree of EGFR activity alteration required to achieve beneficial effects. To date, no EGFR-targeted agents have entered neurological clinical trials. EGFR signaling has been explored mainly in preclinical models of brain injury and neuroinflammation. Furthermore, most available studies rely on acute injury models or transgenic animal models, and their translational relevance to human disease remains unclear.

Optimal dosing regimens are largely unexplored. Key controversies include whether EGFR should be stimulated (to harness its neurotrophic functions) or blocked (to reduce maladaptive gliosis), where evidence exists for both approaches (White et al., 2011; Romano and Bucci, 2020; Badenes, 2024). The available literature suggests that these apparently opposing strategies may not be mutually exclusive. Instead, they likely reflect distinct biological functions of EGFR signaling. Transient ligand-driven activation may support neurogenesis, remyelination, neuronal survival, and tissue repair, whereas chronic or dysregulated EGFR activation may contribute to neuroinflammation, reactive gliosis, and neurodegeneration. This framework raises the possibility that therapeutic benefit may depend on restoring physiological EGFR signaling rather than simply activating or inhibiting the pathway. Accordingly, future therapeutic approaches may need to selectively enhance regenerative EGFR signaling while simultaneously limiting pathological signaling in reactive glial or inflammatory cells. Patient heterogeneity will also be important. For example, EGFR inhibition may prove beneficial in disorders characterized by chronic neuroinflammation and pathological protein aggregation, whereas enhancing EGFR signaling may be more appropriate in conditions where regenerative capacity is impaired, such as demyelinating diseases. Identifying biomarkers that distinguish these disease states and define EGFR signaling status in individual patients will therefore be critical for patient stratification and treatment selection. Safety remains a major concern because EGFR is a proto-oncogene, and chronic pathway activation may increase the risk of tumorigenesis. Conversely, prolonged inhibition can impair endogenous repair mechanisms.

Directly administering EGFR ligands (EGF, TGF-*α*, HB-EGF) may hold therapeutic potential for neurodegenerative diseases, but may depend on co-administration of other growth factors (White et al., 2011; Hernández-Bernal et al., 2024; Sun et al., 2010; Türeyen et al., 2005). Accordingly, EGF combined with growth hormone-releasing hexapeptide in a phase I/II clinical trial for acute ischemic stroke was found safe and led to favorable neurological and functional evolution and higher survival rates, supporting a Phase III study (Hernández-Bernal et al., 2024). Although stroke is not a neurodegenerative disease, these findings provide proof-of-principle that therapeutic manipulation of EGFR signaling in the CNS is feasible. Major challenges include ligand stability, controlling the duration and magnitude of receptor activation, and minimizing oncogenic risk. Potential solutions include viral vectors, engineered peptides, nanoparticles, and cell-based delivery systems designed for localized ligand exposure. Such targeted delivery strategies may also reduce systemic toxicity by restricting EGFR activation to CNS injured or degenerating regions.

In neurodegeneration models, blocking EGFR with tyrosine kinase inhibitors (TKIs) has shown promise. Blood brain barrier (BBB) penetration and dosing/timing are critical issues. Osimertinib has the highest BBB penetration (Choi et al., 2023), while AZD3759, a CNS-penetrant EGFR inhibitor, has demonstrated brain bioavailability and clinical activity (Yang et al., 2016). Other TKIs exhibit limited and variable BBB penetration. Despite encouraging preclinical findings, evidence supporting the use of EGFR inhibitors in neurodegenerative disease remains limited, and no agent has yet demonstrated efficacy in clinical neurological settings. Furthermore, EGFR TKIs (e.g., erlotinib, gefitinib, afatinib) are associated with common toxicities such as rash, and diarrhea during chronic use in cancer patients. Rare but serious adverse effects include interstitial lung disease and, less commonly, renal complications (Li et al., 2024). These safety considerations are particularly relevant when considering use in neurological settings, where long-term tolerability is critical, and may necessitate exploration of lower-dose or intermittent dosing strategies. In addition, because many neurological disorders require prolonged treatment, balancing efficacy with chronic safety will be particularly important when repurposing oncology-derived EGFR inhibitors. EGFR-blocking antibodies do not cross the BBB readily, but can be delivered intrathecally in theory. Their large size and immunogenicity are hurdles. Antibodies could be engineered (e.g., bispecific for BBB transport) to improve CNS delivery.

An emerging alternative is the selective targeting of individual components of the EGFR signaling network rather than global inhibition or activation of the receptor itself. Targeting specific ligands offers selectivity with fewer side effects. Neutralizing antibodies and RNA-based strategies targeting AREG are under preclinical and clinical development for fibrotic diseases (Schramm et al., 2022; Son et al., 2023). By blocking AREG-driven profibrotic signaling while preserving other ligands such as EGF and HB-EGF, such strategies may offer more precise control of tissue repair and pathological scarring. Extension of this concept to neuroinflammatory conditions remains speculative but mechanistically plausible. Inhibition of ADAM10 or ADAM17 or blocking ADAM17 regulators iRhoms and iTAP/FRMD8 are also possible approaches that can be therapeutically explored to limit EGFR activation. Because these interventions influence ligand availability rather than receptor activity itself, they may offer greater flexibility in shaping EGFR signaling outputs. Similarly, selectively modulating specific downstream signaling pathways rather than EGFR itself may eventually provide a means of preserving regenerative signaling while minimizing pathological responses. Finally, combinatorial approaches that integrate EGFR modulation with anti-inflammatory, neuroprotective, or remyelinating therapies may prove more effective than targeting EGFR signaling alone. Future therapeutic strategies will likely require cell-type-specific and temporally controlled modulation of EGFR signaling to maximize regenerative benefits while minimizing pathological consequences. Overall, the evidence reviewed here suggests that the future of EGFR-based therapy is unlikely to rely on simple receptor activation or inhibition, but rather on precision modulation of the EGFR signaling network tailored to disease stage, cellular context, and the underlying pathological mechanisms.

## 2 Discussion

The available literature indicates that EGFR signaling may occupy a pivotal position linking neuronal injury responses, neuroinflammation, and neurodegeneration. A central theme emerging is that the biological consequences of EGFR activation are highly dependent on the timing, magnitude, duration, cellular localization of signaling, and downstream pathway bias. Rather than acting as a purely beneficial or detrimental pathway, EGFR appears to function as a context-dependent regulator of CNS homeostasis, repair, and degeneration. This context dependency may explain many of the seemingly contradictory findings reported across models of neural injury and neurodegenerative disease. Furthermore, heterodimerization of EGFR with ErbB2, ErbB3, and particularly ErbB4 can alter downstream signaling outputs. Among these receptors, ErbB4/Neuregulin1 has by far the strongest evidence in CNS disease. Dysregulation of this pathway is strongly linked to schizophrenia, and is increasingly implicated in AD, epilepsy, and other neurodegenerative disorders (Turner-Ivey et al., 2025; Ding et al., 2024). Collectively, these observations suggest that EGFR should be viewed as part of a broader ErbB signaling network rather than as an isolated receptor, and that interactions among ErbB family members may substantially influence disease-specific biological outcomes.

In neural injury and neurodegenerative diseases, transient EGFR activation promotes neuronal survival, neural stem/progenitor cell proliferation, synaptic plasticity, and tissue repair, whereas chronic or dysregulated activation drives persistent neuroinflammation, reactive gliosis, glial scar formation, and neuronal dysfunction and degeneration (Romano and Bucci, 2020; Badenes, 2024). We hypothesize that the transition from reparative to pathogenic EGFR signaling is governed by a series of interconnected switches, including signal duration, receptor trafficking dynamics, crosstalk with inflammatory, fibrotic and mechanotransductive pathways, and cell-type-specific signaling responses. We speculate that acute EGFR activation generates transient ERK signaling pulses together with temporally restricted PI3K/AKT activation that promote regenerative transcriptional programs and neuronal survival. However, prolonged ligand exposure or impaired receptor trafficking may sustain EGFR mediated ERK/MAPK, JAK/STAT3, and maladaptive PI3K/AKT/mTOR activity in astrocytes and microglia, driving reactive gliosis, chronic cytokine production, and metabolic reprogramming, and contributing to aberrant activation of cell-cycle-associated transcriptional programs (Crozet and Levayer, 2023; Schultz et al., 2023; Patani et al., 2023; Qu et al., 2012; Chu et al., 2021; Liu et al., 2006; Herrup, 2012). Concomitant epigenetic remodeling may stabilize EGFR-driven neuroinflammatory and reactive glial phenotypes long after the initiating stimulus has diminished (Scholz et al., 2024). In addition, injury-induced inflammatory and profibrotic mediators, including TNF-*α*, IL-1*β*, IL-6, and TGF-β, may converge with and potentiate EGFR signaling to amplify ERK/MAPK- and STAT3-dependent transcription, promoting cytokine production, extracellular matrix remodeling, and reactive gliosis. We speculate that persistent crosstalk among these pathways establishes a feed-forward network that reinforces chronic neuroinflammation and pathological tissue remodeling (Liu et al., 2006; Patani et al., 2023; Chu et al., 2021; Meng et al., 2016). Furthermore, injury-driven YAP/TAZ activation may potentiate EGFR signaling by increasing the expression of EGFR ligands, EGFR-associated transcriptional programs, and other growth-factor signaling molecules, thereby establishing a positive feedback loop linking extracellular matrix remodeling and mechanotransduction to regenerative and inflammatory responses (Luo et al., 2025; Zhang et al., 2009; Vigneswaran et al., 2021). In neurodegenerative disorders, chronic exposure to protein aggregates and tissue stress signals may co-opt EGFR-associated signaling networks, shifting EGFR-associated signaling networks toward a persistent STAT3-dependent reactive astrocyte state that contributes to synaptic dysfunction, neuronal loss, and disease progression (Liu et al., 2006; Patani et al., 2023). Although several aspects of this model remain speculative, it provides a mechanistic framework that reconciles the apparently opposing effects of EGFR activation reported across different experimental systems and disease contexts.

Consequently, future studies should move beyond viewing EGFR as a single therapeutic target and instead focus on defining how EGFR signaling operates within specific cellular populations and disease stages. Emerging technologies such as single-cell transcriptomics, spatial transcriptomics, lineage tracing, spatial proteomics, and better translational models, including conditional cell-specific genetic models, will be particularly valuable for addressing these questions. Another gap is long-term safety: most neural repair studies are short-term. Chronic EGFR modulation could have unanticipated effects. Nevertheless, modulating EGFR offers therapeutic promise: delivering EGFR agonists (for instance, by administering EGF-like ligands) can enhance neurogenesis and neuronal survival, whereas EGFR inhibitors (e.g., with small-molecule inhibitors) can dampen neurotoxic pathology in diseases like AD, PD and ALS. Recent preclinical studies in rodents and zebrafish have established proof-of-concept for both approaches. Which strategy is preferable likely depends on disease timing, and cell and disease types. The field now faces the challenge of translating these insights: designing ligand-specific or context-dependent EGFR-targeted therapies that tip the balance toward healing. From a translational perspective, the current evidence suggests that therapeutic modulation of EGFR should be guided by the predominant biological process rather than by disease diagnosis alone. Conditions characterized by impaired regeneration, loss of neural progenitors, or demyelination may benefit from transient and spatially restricted EGFR activation to enhance neuronal survival, oligodendrocyte lineage expansion, remyelination, and tissue repair. In contrast, disorders dominated by chronic neuroinflammation, reactive astrogliosis, pathological protein aggregation, or glial scar formation may be more amenable to selective EGFR inhibition, particularly during later stages of disease when persistent EGFR signaling becomes maladaptive. These strategies should not be regarded as mutually exclusive but rather as complementary approaches that target distinct phases of disease progression. We therefore propose a conceptual therapeutic framework in which the optimal intervention depends on the timing of treatment, the predominant responding cell populations, and the balance between regenerative and pathogenic EGFR signaling. Future therapeutic approaches will likely require biomarker-guided, cell-specific, and temporally controlled modulation of EGFR activity rather than sustained global activation or inhibition. Such precision approaches may ultimately enable preservation of the physiological functions of EGFR while selectively suppressing pathological signaling pathways that drive disease progression. Given the dual nature of EGFR, personalized approaches (targeting EGFR only in appropriate cell types/times) will likely be necessary.

Overall, the evidence reviewed here supports a broader view of EGFR, moving beyond its role as simply a therapeutic target to recognizing it as a dynamic signaling hub whose biological effects are dictated by cellular context, disease stage, and network interactions. Harnessing this complexity, rather than circumventing it, may provide new opportunities for developing effective EGFR-based therapies for neurological disorders.

In summary, EGFR and its ligands are central regulators of CNS cell fate and repair. Leveraging this pathway for neurodegenerative diseases holds promise but requires nuanced understanding of cell-specific signaling and rigorous control of treatment parameters.

## Glossary

AD: Alzheimer’s disease
ADAM17: A Disintegrin and Metalloproteinase 17
ADAMs: A Disintegrin and Metalloproteinases
AKT: protein kinase B (PKB)
ALS: Amyotrophic lateral sclerosis
AREG: Amphiregulin
BBB: Blood brain barrier
BTC: Betacellulin
CNS: Central nervous system
EAE: Experimental allergic encephalomyelitis
EGF: Epidermal growth factor
EGFR: Epidermal growth factor receptor
EPGN: Epigen
EREG: Epiregulin
ERK: Extracellular signal-regulated kinase
FRMD8: FERM-domain containing 8
HB-EGF: Heparin-binding EGF-like growth factor
HD: Huntington’s disease
iRhoms: Rhomboid family of intramembrane pseudoproteases
iTAP: iRhom tail-associated protein
JAK: Janus kinase
JNK: c-Jun N-terminal kinase
MAPK: Mitogen-activated protein kinase
MEK: Kinases-mitogen-activated protein kinase kinase
mTOR: Mammalian target of rapamycin
MS: Multiple sclerosis
PD: Parkinson’s disease
PI3K: Phosphoinositide 3-kinases
PKB: Protein kinase B
PKC: Protein kinase C
PLCγ: Phospholipase C gamma
PNS: Peripheral nervous system
RAF: Proteins-rapidly accelerated fibrosarcoma
RAS: Rat sarcoma vírus
SAH: Subarachnoid hemorrhage
SCI: Spinal cord injury
STAT: Signal transducer and activator of transcription
SVZ: Subventricular zone
TACE: TNF*α* converting enzyme
TGF-α: Transforming growth factor-α
TKI: Tyrosine kinase inhibitor
VEGF: Vascular endothelial growth factor
VEGFR: Vascular endothelial growth factor receptor
